# Pilot randomised controlled trial of focused narrative intervention for moderate to severe depression in palliative care patients: DISCERN trial

**DOI:** 10.1177/0269216317711322

**Published:** 2017-06-07

**Authors:** Mari Lloyd-Williams, Christopher Shiels, Jacqueline Ellis, Katharine Abba, Edward Gaynor, Kenneth Wilson, Christopher Dowrick

**Affiliations:** 1 Academic Palliative and Supportive Care Studies Group, Institute of Psychology, Health and Society, University of Liverpool, Liverpool, UK; 2 Liverpool Clinical Commissioning Group, Liverpool, UK; 3 Division of Psychiatry, Institute of Psychology, Health and Society, University of Liverpool, Liverpool, UK; 4 Primary Medical Care, Institute of Psychology, Health and Society, University of Liverpool, Liverpool, UK

**Keywords:** Depression, advanced cancer, palliative care, hospice, Patient Health Questionnaire-9, focused narrative intervention, randomised controlled trial

## Abstract

**Background:**

Depression is poorly detected and sub-optimally managed in palliative care patients, and few trials of psychosocial interventions have been carried out in this group of patients.

**Aims:**

A pilot trial to determine the effect of a focused narrative intervention on depression in palliative care patients when used in addition to usual care.

**Design:**

Patients scoring 10 or higher on Patient Health Questionnaire-9 randomised to focused narrative intervention in addition to usual care or usual care only and followed up at 2, 4 and 6 weeks. A reduction of five points on Patient Health Questionnaire-9 was regarded as clinically significant response to treatment.

**Setting/participants:**

Palliative care patients aged over 18 recruited from hospice day care services – exclusion criteria included an estimated prognosis of 6 weeks or less, cognitive impairment and unable to understand written or spoken English.

**Results:**

Out of 57 participating patients (71% female), with mean age 65.1 years (range 36–88 years), 33 patients were randomised to the intervention and 24 to usual care only. Mean Patient Health Questionnaire-9 score at baseline was 16.4. Patients receiving intervention had greater reduction in Patient Health Questionnaire-9 score at 6-week follow-up (*p* = 0.04). Median survival was 157 days for intervention and 102 days for control group patients (*p* = 0.07).

**Conclusion:**

This pilot trial suggests a focused narrative intervention in palliative care patients with moderate to severe depression can reduce depression scores more than usual care alone. Patients receiving intervention appeared to have longer survival. These results support the need for a fully powered trial.

**What is already known about the topic?**

- Depression is common in the palliative care population.
- The evidence for effective interventions for depression is limited.

**What this paper adds?**

- Focused narrative intervention can be an effective intervention for moderate to severe depression in palliative care patients when used in addition to usual care.
- The effect of the intervention appears to be sustained at 6-week follow-up.
- Those randomised to the intervention appeared to have longer survival than patients randomised to usual care.

**Implications for practice, theory or policy**

- This study supports the requirement for a larger randomised controlled trial.
- The focused narrative intervention could be delivered by any member of a palliative care team.
- The cost benefits of the intervention need to be explored in further studies.

## Introduction

Depression in palliative care patients is common, difficult to assess, and treatment is complex.^1^ There are several known risk factors for depression in those with cancer, including younger age and a history of receiving psychological treatment.^2,3^ Depression impairs quality of life and can cause earlier mortality in palliative care populations.^4–7^ European guidelines support rigorous assessment and management of depression in patients with advanced cancer.^8^

Treatment of depression includes use of pharmacological and psychological interventions. Antidepressants influence recovery from depression in patients with advanced disease,^1^ and findings of a meta-analysis suggested that antidepressants were superior to placebo and effect increased with continued use.^9^ One of the earliest studies of non-pharmacological interventions^10^ found that group support significantly improved psychological well-being in metastatic breast cancer, and group therapy has been found to reduce new episodes of depression in women with metastatic breast cancer.^11^

Much of the focus of interventions for depression has been with patients with early disease, and there is a dearth of psychological interventions created specifically for palliative care patients. In a trial of 80 palliative care patients, cognitive behavioural therapy had an effect on anxiety but no effect was observed on depression.^12^ Chochinov et al.,^13^ in an initial study of 100 palliative care patients using Dignity therapy, reported a reduction in depressive symptoms. However, a subsequent trial^14^ revealed no significant differences in depression scores post-intervention as measured by the Hospital Anxiety and Depression (HAD) scale. More recently, meaning-centred group psychotherapy has been found to improve psychological well-being in advanced cancer^15^ as has the individual integrated collaborative care model^16^ and other individual interventions have also been developed.^17–19^

Within a clinical setting, patients often report that they ‘feel better’ at the end of a clinical consultation when the only intervention has been to give patients the necessary space and cues to allow them to tell their illness story. Narrative therapy techniques are particularly effective for people with cancer. It has been reported that ‘narrative therapy as an intervention makes an important contribution to the holistic support of the dying patient and his or her family’ with a number of therapeutic benefits, including to objectify and distance oneself from problems in order to gain understanding, establish meaning, develop greater knowledge and to decrease emotional distress.^20,21^ Narratives offer the opportunity to express feelings without having to worry about their effect on others and can promote coping strategies. Narratives can offer a safe place for individuals to explore the implications of their experiences.^22^ In a trial with older adults, it was found to reduce depressive symptoms over a follow-up of 9 months and, additionally, to reduce anxiety^23^ and to reduce depressive symptoms in palliative care patients.^24^

We report the findings of a pilot trial developed from a programme of work based on the Medical Research Council (MRC) framework for the development and evaluation of complex interventions.^25^ The trial piloted a focused narrative intervention to determine if the intervention in addition to usual care, when compared to usual care alone, had an effect on moderate to severe depression (as measured by the Patient Health Questionnaire (PHQ)-9) in palliative care patients with advanced cancer.

## Methods

### Study design

This study was a non-blinded randomised controlled trial of two groups of patients – the patients randomised to the intervention group receiving the focused narrative intervention in addition to usual care and the control group receiving usual care only. Within a hospice setting, usual care would include antidepressant medication and access to complementary therapies and counselling.

### Participants and settings

The study was carried out in six hospice day units in the North West of England – recruitment into the study commenced on 1 May 2013 and ended on 14 December 2015. All new patients older than 18 years with a diagnosis of advanced cancer, attending hospice day care services, with an Eastern Cooperative Oncology Group (ECOG)^26^ performance of one or two, were invited to participate in the study. All hospice services accepted referrals for patients with advanced life-limiting cancer with a life expectancy of 12 months or less. The only specific exclusion criteria for the trial were severe cognitive impairment that would impede consent (based on clinical judgement), insufficient understanding of English and a prognosis of 6 weeks or less.

### Depression outcome measures

The PHQ-9 and a patient-rated outcome measure (PROM) were administered to patients as part of the baseline assessment and at 2-, 4- and 6-week follow-up. The PHQ-9, the primary outcome in the trial, is a 9-item self-report instrument originally developed to screen for major depressive disorder in primary care settings. It can be scored continuously as a measure of symptom severity or with validated cut offs for mild (score = 5–9), moderate (score = 10–14), moderately severe (score = 15–19) and severe depression (score = 20–27)^27,28^ and has been validated in cancer populations.^29,30^ A threshold score of 10 can be used to identify a depression ‘case’. A single item PROM was also included in the trial, requesting the patient to indicate whether they felt ‘very depressed’, ‘quite depressed’, ‘a little depressed’ or ‘not depressed at all’.

#### The Edmonton Symptom Assessment was administered at baseline

The Edmonton Symptom Assessment (ESAS)^31^ is a valid and reliable assessment tool to assist in the assessment of nine common symptoms experienced by cancer patients. This tool is designed to assist in the assessment of pain, tiredness, nausea, depression, anxiety, drowsiness, appetite, well-being and shortness of breath. The severity at the time of patient assessment of each symptom is rated from 0 to 10 on a numerical scale; with 0 meaning that the symptom is absent and 10 that it is the worst possible severity. An additional item on ‘will to live’ has been added to the core nine ESAS scales.

### Other patient information collected

Patient age and gender were routinely recorded at baseline assessment. A neighbourhood social deprivation score (the lower layer super output area score in the 2015 Indices of Deprivation) was generated from the post code of each patient.

### Procedure

Eligible patients were informed of the study by letter. Patients who agreed to be contacted by a researcher received full details regarding the study. Following consent, patients were invited to complete baseline assessment tools which patients completed unaided. Patients who scored 10 or above on the PHQ-9 (indicative of moderate to severe depression) at baseline assessment, and who consented to participate in the trial, were allocated to a trial arm (intervention plus usual care or usual care only) by means of randomly allocated opaque envelopes. Follow-up questionnaires were completed at 2, 4 and 6 weeks following the delivery of the intervention and baseline data collection in the usual care only arm. Any patients who expressed any positive response to the PHQ-9 question regarding suicidal ideation were referred onto the hospice team and managed according to usual hospice practice. Full ethical approval for the study was obtained (Reference 13/NW/0203).

### Intervention

Patients randomised to the intervention arm were offered, in addition to usual care, a focused narrative intervention. The intervention was developed from the literature reviews and expert clinician consensus and had been piloted and refined. A single semi-structured narrative face to face interview was carried out by trained researchers with a health background and experience in research with patients with advanced illness. The intervention was delivered either in the hospice or the patient’s home. The interviews were, if possible, conducted within a week of the baseline data collection and of patients being randomised. The researcher prompted the patients to discuss perspectives on their sense of meaning regarding distress/depression and their physical, psychological and spiritual well-being, the emphasis being on allowing patients to share their thoughts and experiences in a supportive environment. Patients were encouraged to share what they felt had been the main contributing factor for depression and distress and to share what resources they themselves had employed in addition to any medical/professional care received, with emphasis being on helping patients to reflect on their own inner resources and coping methods. The pace, sequencing and duration of the interviews depended on the patients, with interviews lasting from 25 to 60 min. A random selection of interviews was monitored by the chief investigator to ensure a consistent approach and to maintain fidelity.

### Analysis

Any differences in the composition of the two trial groups were assessed by the application of the *t*-test for continuous variables. Significance of differences in proportions was assessed by the chi-square test and the Cochran–Armitage test for trend. The 95% confidence intervals (CIs) of means and proportions were also reported. For the calculation of the latter, the Newcombe–Wilson method was used. The significance of the inter-group difference in mean PHQ-9 score at baseline, and at each of the three follow-up points, was tested by the *t*-test. The interaction between change in mean scores across the four measurement points and membership of a trial arm was assessed by a repeated measures analysis of variance (ANOVA).

The inter-group difference in proportions of patients in the PROM categories (not/little/quite/very depressed), at baseline, was tested by the chi-square. For looking at the significance of change over time between the intervention and control group, the PROM ordinal scale was treated as interval data, and a repeated measures ANOVA was run.

Analysis of covariance (ANCOVA) was performed to test for significant changes in PHQ-9 score from baseline to 2-, 4- and 6-week follow-up for residual members of the control and intervention groups. For the PROM, significance of inter-group differences from baseline to each of the follow-up points was tested by the use of the Wilcoxon signed-rank test. For all inter-group comparisons, the CI of difference in means/proportions is reported, along with the estimate of statistical significance.

All trial participants were depression ‘cases’ (PHQ-9 score ⩾ 10) at baseline, a random-effects logistic regression model was run in order to estimate the effect of trial group membership on likelihood of the patient being a ‘non-case’ (PHQ-9 score < 10) at their final follow-up measurement point. Including follow-up time point (2, 4 and 6 weeks) as an intercept in the multilevel model allowed the inclusion of patients who may not have completed the PHQ-9 at all three follow-up points. The estimated effect of the trial arm on likelihood of outcome was adjusted for baseline differences in the composition of the intervention and control groups. Odds ratio (OR), 95% CI, and associated *p* value are reported.

Kaplan–Meier survival curves were mapped for control and intervention group patients. Mortality rate and median time to death are reported. The Mann–Whitney *U* test was used to test whether there were significant differences in time to death between the two trial groups.

For all analyses, a conventional criterion of statistical significance (*p* < 0.05) was applied. All data were analysed using SPSS for Windows 22.0 and STATA IC 11.

## Results

**Figure 1.**
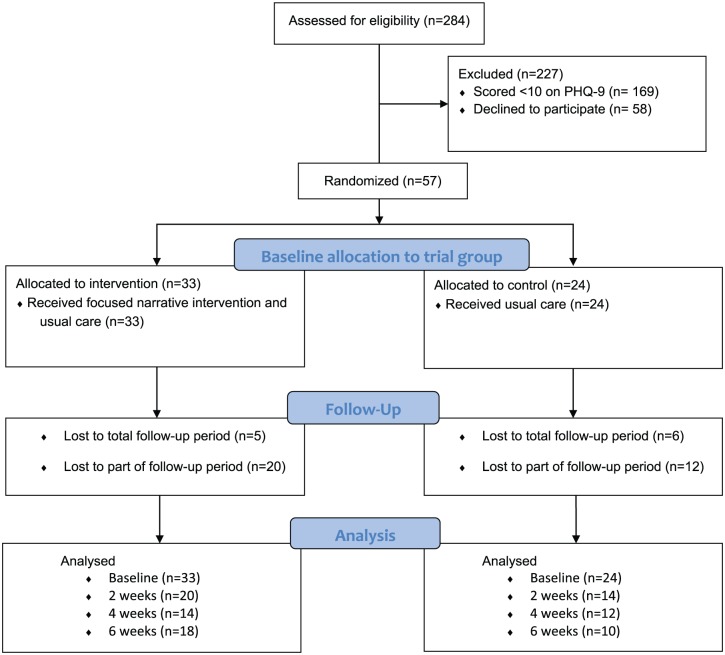
Patients in DISCERN trial.

In total, 284 patients were screened for the trial (mean age = 66.7 years; range = 27–94 years), and 63.7% were female (Figure 1). Of these, 169 (59.5%) scored less than 10 on PHQ-9 and were ineligible to participate in the trial. A total of 58 declined to take part in the trial. Of the remaining 57 patients, 33 were randomised to the intervention group and 24 to the usual care group only (Table 1). The majority, 39 (71%), were female, and the mean age was 65.1 years (range = 36–88 years, median age = 66 years). The mean PHQ-9 score at baseline was 16.4, and 16% indicated that they were ‘very depressed’ on the Patient Reported Outcome Measure. A fifth of patients in the trial had a post code within the 20% most deprived neighbourhoods in England. All trial participants scored 1 or 2 on the ECOG measure of performance status.^26^ In total, 19 (57.3%) of the 33 patients in the intervention group and 11 of the 24 (45.8%) patients in the control group were currently prescribed antidepressant medication.

**Table 1.** Composition of control and intervention groups at baseline.

	Control	Intervention	*p*
% female	65.2 (95% CI: 44.9–81.2)	75.0 (95% CI: 57.9–86.7)	0.43
Mean age of trial group members	63.4 (95% CI: 59.2–67.6)	66.2 (95% CI: 62.1–70.3)	0.40
% living in one of 20% most deprived LSOAs in England	13.0 (95% CI: 4.5–32.1)	25.8 (95% CI: 13.7–43.2)	0.25
Type of cancer diagnosis (%)			
Breast	26.2 (95% CI: 12.5–46.5)	16.7 (95% CI: 7.3–33.6)	0.15
Gastrointestinal	4.3 (95% CI: 0.2–2.1)	10.0 (95% CI: 3.5–25.6)	
Lung	21.7 (95% CI: 9.7–41.9)	6.7 (95% CI: 1.8–21.3)	
Head/neck	8.7 (95% CI: 2.4–26.8)	0 (–)	
Male-specific	8.7 (95% CI: 2.4–26.8)	6.7 (95% CI: 1.8–21.3)	
Female-specific	8.7 (95% CI: 2.4–26.8)	6.7 (95% CI: 1.8–21.3)	
Other	21.7 (95% CI: 9.7–41.9)	53.2 (95% CI: 36.1–69.8)	
Mean PHQ-9 score	14.6 (95% CI: 13.1–16.1)	17.6 (95% CI: 16.4–18.8)	0.005
% of trial group reporting as ‘very depressed’ on PROM	8.3 (95% CI: 2.3–25.8)	21.9 (95% CI: 11.0–38.8)	0.33
% on antidepressant medication	45.8 (95% CI: 27.9–64.9)	57.6 (95% CI: 40.8–72.8)	0.38
Mean ESAS ratings (higher = increasing negative experience)			
Pain	4.7 (95% CI: 3.7–5.7)	5.7 (95% CI: 4.9–6.3)	0.12
Tiredness	7.3 (95% CI: 6.3–8.3)	7.5 (95% CI: 6.9–8.1)	0.72
Nausea	3.0 (95% CI: 1.8–4.2)	2.1 (95% CI: 1.1–3.1)	0.24
Depression	5.3 (95% CI: 4.3–6.3)	6.9 (95% CI: 6.4–7.4)	0.02
Anxiety	5.8 (95% CI: 4.8–6.8)	6.2 (95% CI: 5.2–7.2)	0.54
Drowsiness	5.7 (95% CI: 4.5–6.9)	6.3 (95% CI: 5.5–7.1)	0.44
Appetite	5.1 (95% CI: 4.1–6.1)	5.5 (95% CI: 4.5–6.5)	0.61
Well-being	5.4 (95% CI: 4.7–6.1)	6.5 (95% CI: 5.8–7.2)	0.07
Shortness of breath	4.0 (95% CI: 2.8–5.2)	5.4 (95% CI: 4.2–6.6)	0.15
Will to live	3.3 (95% CI: 2.1–4.5)	3.8 (95% CI: 2.6–5.0)	0.54

CI: confidence interval; LSOA: lower layer super output areas; PHQ: Patient Health Questionnaire; PROM: patient-rated outcome measure; ESAS: Edmonton Symptom Assessment.

### Baseline and follow-up PHQ-9 scores

All patients completed a PHQ-9 at baseline; 34 (59%) completed 2-week follow-up, 26 (45%) completed 4-week follow-up and 28 (59%) completed 6-week follow-up. Total study attrition was 49% (45% in the intervention arm and 58% in the control group). A total of 14 patients (24.5%) (8 intervention and 6 control) completed PHQ-9 at all time-points.

The intervention group had a significantly higher mean PHQ-9 score than the control group at baseline (17.6 vs 14.6, *p* = 0.005). There was a trend for PHQ-9 scores to reduce for all participants during the 6-week follow-up period. A reduction of five points in PHQ-9 is defined as being a clinically significant response to depression treatment,^32^ and at 2 weeks, 10 of the intervention group (50%) had a reduction in PHQ-9 scores of five or more compared to 4 of 14 (28.6%) of the control arm (*p* = 0.21). At 4 weeks, 7 of the 14 intervention group patients (50%) had a reduction in PHQ-9 scores of five points or more compared to three (25%) of the control arm (*p* = 0.19). At 6 weeks, 11 of the intervention group who completed follow-up (61.1%) had an improvement of five points or more in PHQ-9 score compared to two (20%) of the control group (*p* = 0.04). Patients in the intervention group showed a greater reduction in PHQ-9 scores than control group at each follow-up; however, this was not statistically significant (*p* = 0.25) (Table 2).

**Table 2.** Mean PHQ-9 scores and mean changes in scores for patients in control and intervention groups.

	PHQ-9 score at baseline (*C* = 24; *I* = 33)	PHQ-9 score at 2 weeks (*C* = 14; *I* = 20)	PHQ-9 score at 4 weeks (*C* = 12; *I* = 14)	PHQ-9 score at 6 weeks (*C* = 10; *I* = 18)	*p*
	Mean (95% CI)	Mean (95% CI)	Mean (95% CI)	Mean (95% CI)	
Control	14.6 (13.2 to 16.0)	13.4 (10.7 to 16.1)	12.3 (9.7 to 14.9)	10.1 (7.7 to 12.5)	0.25
Intervention	17.6 (16.3 to 19.0)	12.6 (9.7 to 15.5)	12.3 (10.1 to 14.6)	13.9 (11.2 to 16.6)	
95% CI of difference in means	(1.13 to 5.21)	(−5.18 to 3.52)	(−3.57 to 3.64)	(−0.48 to 8.17)	
*p*	0.005	0.70	0.98	0.08	

PHQ: Patient Health Questionnaire; CI: confidence interval.

A higher proportion of the intervention group patients who completed a PROM rating at 4 weeks reported their depression status had improved compared to those of the usual care group (Table 3).

**Table 3.** Improvement in PHQ-9 score and PROM depression rating between baseline and follow-up points.

	⩾5 point reduction in PHQ-9 score (%)	% rating depression status as improved on PROM
	(95% CI)	(95% CI)
Base to 2 weeks		
Control (*n* = 14)	28.6	28.6
Intervention (*n* = 20)	50.0	22.2
95% CI of difference	(−11.5 to 47.6)	(−36.0 to 22.2)
	*p* = 0.21	*p* = 0.68
Base to 4 weeks		
Control (*n* = 12)	25.0	33.3
Intervention (*n* = 14)	50.0	46.2
95% CI of difference	(−11.5 to 53.2)	(−23.1 to 44.3)
	*p* = 0.19	*p* = 0.51
Base to 6 weeks		
Control (*n* = 10)	20.0	40.0
Intervention (*n* = 18)	61.1	31.3
95% CI of difference	(2.8 to 64.2)	(−42.2 to 24.9)
	*p* = 0.04	*p* = 0.48

PHQ: Patient Health Questionnaire; PROM: patient-rated outcome measure; CI: confidence interval.

### The effect of intervention on depression status

A total of 45 patients (24 control and 33 intervention) were followed up at least once. As reported above, all patients at baseline were depression cases (PHQ-9 score ⩾ 10). A random-effects logistic regression model was run in order to estimate the effect of receiving the intervention on change in depression status, that is, having a non-case depression status at end of the follow-up period (or at final completion of a PHQ-9). The effect of trial group membership on outcome was adjusted for group differences in baseline PHQ-9 score and antidepressant medication use, and the number of patient follow-up measurements.

Compared to those in the control arm, patients receiving the intervention had raised (but not statistically significant) odds of becoming a depression non-case at time of leaving or completing the study (OR = 1.78, 95% CI: 0.44, 7.24, *p* = 0.42).

### Survival

**Figure 2.**
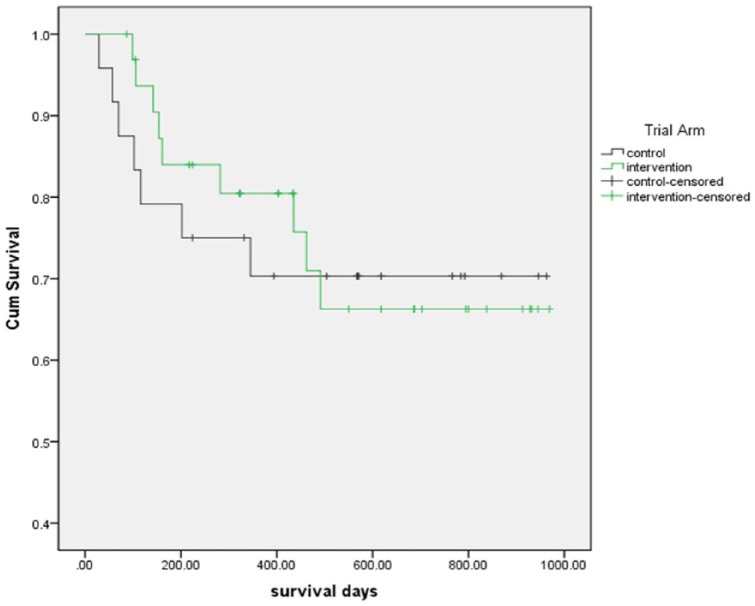
Survival curves for control and intervention group patients.

A total of 16 trial participants (9 in the intervention group and 7 in the control group) died. Kaplan–Meier survival curves are presented in Figure 2 (Log-rank: *p* = 0.88). Median survival for all participants was 142 days (range = 10–491 days). For patients in the intervention group, the median survival was 157 days compared to 102 days for control group patients (*p* = 0.07).

### Discussion

Depression is known to be common in palliative care patients, and in this study, 41% of 284 patients that were screened scored 10 or more on PHQ-9 and were eligible to participate in the trial. This pilot trial revealed PHQ-9 scores reduced in both arms of the trial over the 6-week follow-up. A reduction of five points in PHQ-9 is defined as being a clinically significant response to depression treatment, and at each follow-up time point, a greater proportion of patients in the intervention group had a reduction in PHQ-9 scores of five points or more than those in the control group. Patients in the intervention group were more likely to have become depression non-cases by the end of follow-up. We used a patient reported outcome tool as we believed it was important, to explore how patients themselves experienced depression and whether they felt an improvement. At 4 weeks, a greater proportion of patients in the intervention group rated their depression as improving compared to the care as usual group.

This study is unique in that we were trialling an intervention to help palliative care patients with moderate to severe depression and the mean PHQ-9 score of patients in the trial was 16.4. Other studies recruiting palliative care patients into trials of non-pharmacological interventions for depression have recruited patients with lower threshold entry scores for depression, for example, threshold of 8 for depression on HAD scale^12^ and HAD depression scores ranging from 5.8 to 6.3 across three arms of an intervention trial.^14^ In a recent trial of brief individualised psychotherapy for advanced cancer, the PHQ-9 mean score at baseline was 8.7.^33^

All outcomes used in the analysis were based upon PHQ-9 scores, including an absolute change in scores over time and change in depression status from ‘case’ (PHQ-9 score ⩾ 10) to ‘non-case’ (PHQ-9 score < 10). Because of the small numbers and the rate of attrition, no attempt was made to identify a minimal clinically important difference (MCID). Instead, we used the ⩾5 point difference in PHQ-9 score between baseline and follow-up previously reported.^32^

The patients within the trial were attending hospice day services, and patients of all ages and diagnoses were included in the trial. The trial was conducted with patients who were in the last weeks and months of life. The median time to death was 142 days (range = 10–491 days). Due to the small numbers, it is impossible to make any statistical inferences, and other factors could account for survival differences. A total of 16 patients (28%) died in the study observation period, and the median survival of patients in the intervention group was 157 days compared to 102 days for usual care group (*p* = 0.07). This finding is of interest as survival is rarely included in palliative care studies. Patients randomised to the intervention group had significantly higher PHQ-9 scores than those in the usual care group, and depression causes earlier mortality in patients with advanced cancer;^7^ therefore, these findings are of interest.

## Strengths and limitations

The total study attrition was 49%, with 45% attrition in the intervention group and 58% in the control group, and it is unlikely that the intervention was responsible for the attrition. All of the trial patients were in the last weeks and months of life and many were undergoing palliative treatment, including chemotherapy, radiotherapy and surgery, which accounted for attrition. Additionally, all were suffering from at least two known major co-morbidities (advanced cancer and moderate to severe depression) making compliance in any study challenging. We did not obtain ethical approval to either send reminder follow-up questionnaires or reminder phone calls, which would probably have reduced our rates of attrition. This study recruited advanced cancer patients from hospice day units only; a recent study of a psychotherapeutic intervention which recruited patients with stage 4 cancer from within oncology and palliative care settings reported higher attrition rates of 68%^33^ than those observed in our study. This suggests that although patients in our study were all within the last weeks and months of life, the acceptability of the intervention enabled a greater proportion of patients to be retained within the study.

Our threshold for recruitment into the trial was a PHQ-9 score of 10 or more, but 50% of those eligible did not participate. We had ethical permission to interview patients who did not wish to participate in the trial (results will be published elsewhere), and this revealed that some patients believed that as they were already receiving holistic care and support from the hospice day care team, they should not ask or expect any further help or support for their depression – a finding previously reported in a study of older people with depression.^34^ This issue would be addressed in any subsequent trial via a more detailed explanation of the intervention within patient information sheets at initial screening.

A key strength of this study is that we managed to recruit patients of all ages and diagnostic groups and patients with moderate to severe scores for depression using a well-validated tool, the PHQ-9. We acknowledge that PHQ-9 scores in the intervention group were statistically higher than those in the control group, and more intervention group patients had been prescribed antidepressants. We have attempted to control for these baseline differences within our regression analysis. However, we accept that our results may have been different if recruitment had included oncology clinics or primary care settings and that our trial sample was small with significant attrition.

## Conclusion

Our work suggests that palliative care patients with moderate to severe depression may benefit from a focused narrative intervention in addition to usual care, as evidenced by reduction in scores on PHQ-9. The follow-up of 6 weeks was felt appropriate in patients with limited prognosis, and although depression scores fell in both arms of the trial, a greater number of patients in the intervention group had a reduction of more than five points in PHQ-9 which was sustained at final follow-up. We believe this pilot trial is unique as recruitment was within palliative care settings and our mean score of 16.4 on PHQ-9 at entry into the study is markedly higher than other intervention studies for depression in advanced cancer populations. Many therapeutic interventions trialled within palliative care have not been adopted within clinical practice, as they required professional qualifications to deliver the intervention. This intervention could be delivered by practitioners with health care–related backgrounds and an understanding of both depression and the context of living with a terminal illness. We wish to further explore its utility as a potential cost-effective intervention for depression in this patient group.

## Appendix 1

### Focused narrative intervention

- In an overall sense, do you feel you are depressed?
  - How bad does it get?
  - Is it a problem for you?
  - Does it come and go or do you feel that way all the time?
  - How much does it bother you?
- If you tried to explain to someone what your depression/low mood was like what would you say? Please try to describe it.
- What is the main thing causing you to be depressed?
- Is there anything that has helped to reduce your depression or low mood? What resources have you drawn upon to cope with your depression?
- What has stopped your depression from getting any worse?
- What aspects of your life are most important to you?
  - Has having cancer changed how you think about your life?
- How did you cope with difficult times in your life before you had cancer?
  - Has having cancer changed how you cope with things in your life?
- Does faith or spirituality play a part in your life? In what way? Can you tell me more about that?
- Some people have told us that they find it hard to make sense of what has happened to them – that they feel it’s not fair, or they ask why me? Why now?
- Is hope important for you? What do you hope for? Has this changed since you found out about the cancer?
- What resources have you drawn upon to cope with situations?
